# Wellen's syndrome: An ominous EKG pattern

**DOI:** 10.4103/0974-2700.55347

**Published:** 2009

**Authors:** Nicole E Mead, Kelly P O'Keefe

**Affiliations:** Department of Emergency Medicine, University of South Florida, Florida, USA

**Keywords:** Inverted T-waves, proximal left anterior descending stenosis, Wellens' syndrome

## Abstract

Wellen's syndrome is a characteristic T-wave on an electrocardiogram during a pain-free period in a patient with intermittent chest pain. This finding suggests a high-degree stenosis of the proximal left anterior descending (LAD) coronary artery that will soon result in an acute anterior wall myocardial infarction (MI) if the patient is not urgently catheterized and the occlusion opened. This case report discusses a young male patient with no known cardiac disease with an EKG that demonstrates the classic Wellen's T-waves. He was urgently taken to cardiac catheterization and his 95% proximal LAD stenosis was reduced via drug-eluding stent. Through knowledge of Wellen's T-waves, more anterior wall MIs can be prevented.

## INTRODUCTION

Wellens' syndrome is characterized by classic T-waves found in precordial leads especially V2-V3 during pain-free periods in a patient presenting with chest pain. The classic T-waves are described as either deeply inverted or biphasic. These findings reliably suggest a high-grade stenosis of the proximal left anterior descending (LAD) coronary artery. Discovering Wellens' syndrome is imperative as 75% of patients will develop acute anterior wall myocardial infarctions (MIs) within one week unless intervention is undertaken urgently.[1–3] The case discussed is unique in that the patient had no known cardiac disease and had not experienced any prior chest pain.

## CASE REPORT

A 37-year-old male presented to the emergency department (ED) for new onset of chest pain of 2 days duration. The pain was described as an intermittent pressure, 7 out of 10 in severity, located retrosternally and in the left parasternal region, and radiating down the left arm. Associated symptoms included profuse diaphoresis, dizziness, shortness of breath and palpitations. There was no associated nausea, vomiting or back pain. One day prior to the ED visit, he experienced three episodes of this chest pain. Two occurrences were associated with exertion, lasting for about 15 min and relieved by rest, while the other episode awoke him from sleep. The most recent episode was immediately prior to his ED presentation. The patient was pain-free when the EKG was accomplished.

The patient denied any previous episodes of chest pain. He also denied a history of hypertension, hyperlipidemia or arrhythmia. He did have a 22-pack-year smoking history, and reported occasional binge drinking. He denied any illicit drug use including cocaine. His mother sustained an acute MI in her 50s.

**Figure 1:**
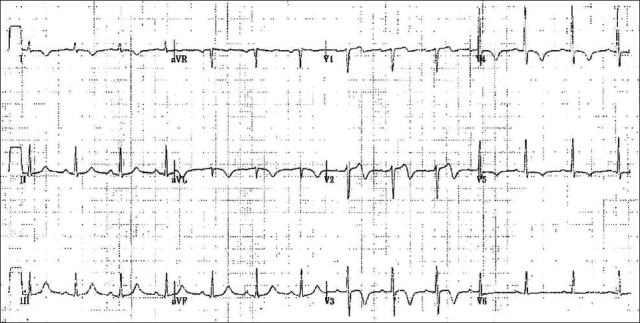
EKG suspicious for Wellen's syndrome with biphasic T-waves in V1 and V2 and deeply inverted T-waves in V3 and V4

On physical examination, the patient was in no distress and was pain-free. Vital signs were: pulse 83, BP 125/80, T 36.6°C, RR 18/min, and saturation on room air was 99%. The cardiovascular examination was normal, as was the remainder of the physical examination. There was no cardiomegaly, infiltrates, effusions or other abnormality seen on chest radiograph. The EKG obtained without pain being present showed normal sinus rhythm, normal axis, narrow QRS, no Q waves, isoelectric ST-segment, biphasic T waves in V1 and V2 and deep, inverted T waves in V3 and V4 [Figure 1].

The EKG was immediately recognized as representing Wellen's syndrome. Cardiac enzymes and lipid profile were obtained and found to be within normal limits. The patient was placed on oxygen via a nasal cannula as well as a cardiac monitor. Aspirin 325 mg was administered, and the patient was started on a nitroglycerin drip. He also received metoprolol. Heparin was held as the patient's stool tested positive for occult blood.

**Figure 2:**
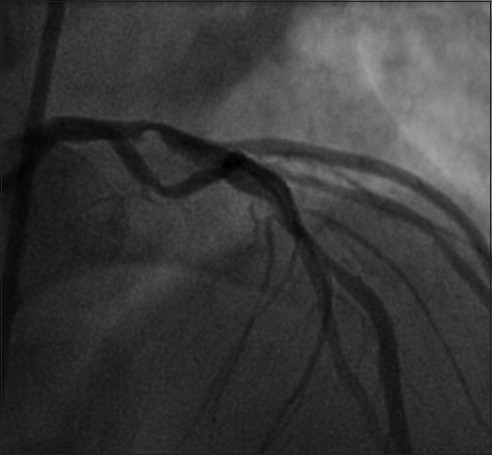
95% stenosis of left anterior descending

The patient was admitted to the CCU and taken to cardiac catheterization the next morning. He was found to have multivessel disease, with the proximal LAD artery severely stenosed with a 95% blockage [Figure 2]. High-moderate disease was discovered in the mid-LAD, proximal left circumflex and ostial right coronary artery. Two drug-eluting stents were deployed, one in the proximal LAD and the other in the D1. The left ventriculogram demonstrated normal left ventricular function with an ejection fraction (EF) of 55-60%. An echocardiogram estimated EF to be 60-65% and found no dysfunction.

The patient was discharged the following day, with no complications and placed on atorvastatin, clopidogrel and aspirin for outpatient therapy].

## DISCUSSION

Wellen's syndrome was first described by Gerson and colleagues in 1980 as an inverted U-wave, and again in 1982 by De Zwaan, Wellens and colleagues as Wellen's syndrome.[1,2] It consists of a characteristic EKG finding suggesting severe stenosis of the proximal LAD artery, which will develop into an acute anterior wall MI within a few days to weeks in 75% of untreated patients.[1–3] Wellen's syndrome is diagnosed based on the classic T-wave findings seen on an EKG taken when the patient is pain-free. These T-wave changes represent reperfusion of the myocardium.[4]

There are two variations of Wellen's syndrome T-wave. Type A is the more common abnormality, occurring in 75% of cases, and is characterized by deeply inverted T-waves in V2 and V3. Type B occurs in 25% of cases and is characterized by biphasic T-waves in V2 and V3.[4,5] The diagnostic leads for T-waves of Wellens' syndrome are V2 and V3, corresponding with a lesion between the first and second septal branches of the LAD. However, if the lesion is more proximal in the LAD, the T-wave changes will be more widely spread along the precordial leads.[4]

**Figure 3:**
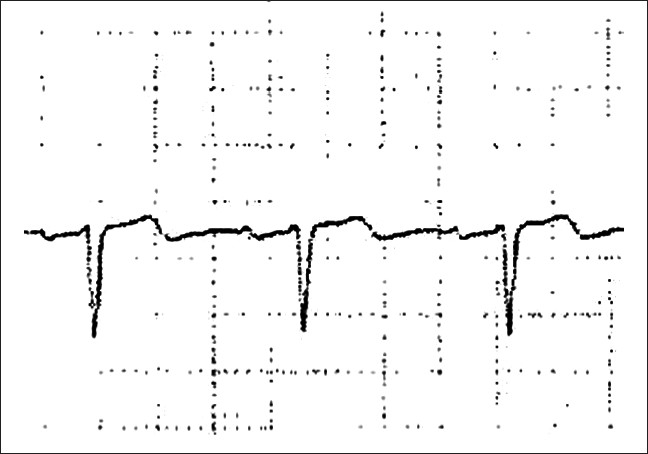
Common T-waves seen in Wellen's syndrome during episode of anginal pain

An EKG obtained during episodes of pain will demonstrate upright T-waves with possible ST segment elevation or depression, but an isoelectric ST segment may also be seen. [5] Cardiac enzymes will be normal or mildly elevated[6] [Table 1]. [5,7] Dr. Wellens describes an essentially normal EKG in patients with critical LAD stenosis with the exception of a slight negative deflection at the end of the T-waves in leads V1 and V2 when anginal pain is present at the time of the EKG [Figure 3].[8] These changes are easily missed, and therefore it is critical for the Emergency Physicians to be aware of them. It is unlikely that any Emergency Physician would miss the deeply inverted T-waves that occur in this syndrome when the patient is pain-free, as is shown in our case, but the significance of these findings must also be recognized. Emergency angiography is justified with either of these EKG presentations, with the hope of avoiding an extensive anterior wall MI through early intervention.[5,9]

**Table 1:** Wellen's syndrome criteria

Prior history of chest pain
During chest pain: EKG is normal or with mild ST elevation or depression, or with terminal negative deflection of the T wave in V_1_ and V_2_
Cardiac enzymes are normal or mildly elevated
No pathologic precordial Q-waves
No loss of precordial R-waves
Deeply inverted or biphasic T-waves in V2 and V3, possibly V1, V4, V5 and/or V6 when pain free

There are some case reports of Wellen's syndrome that do not conform to the classic criteria. One such case described by Riera *et al.*, reported a possible variant of Wellen's syndrome that also presented with left septal fascicular block (LSFB), which is supplied by septal branches of the LAD. Criteria for LSFB are increased R-wave voltage in V2 (R > 15 mm) or V1 (R ≥ 5 mm), normal QRS duration, R/S ratio in V2 > 2 and S-wave depth in V2 < 5 mm.[10]

## CONCLUSION

Wellen's syndrome presents with characteristic EKG findings that all Emergency Physicians need to recognize due to the significant percentage of patients who will develop anterior wall myocardial infarctions if aggressive intervention is not undertaken. Patients in whom Wellen's syndrome is suspected should undergo urgent cardiac catheterization. Stress testing is contraindicated as increasing cardiac demand with a highly stenosed left anterior descending may lead to myocardial infarction. Patients who do not have known heart disease or multiple common cardiac risk factors such as our patient can be spared from an acute myocardial infarction by quick recognition of these classic T-wave changes.

## References

[1] De Zwaan C, Bar FW, Wellens HJ (1982). Characteristic electrocardiographic pattern indicating a critical stenosis high in left anterior descending coronary artery in patients admitted because of impending myocardial infarction. Am Heart J.

[2] Movahed MR (2008). Wellen's Syndrome or Inverted U-waves?. Clin Cardiol.

[3] Smith S, Whitwam S (2006). Acute Coronary Syndromes. Emerg Med Clin N Am.

[4] Nisbet B, Zlupko G (2008). Repeat Wellen's syndrome: Case report of critical proximal left anterior descending artery restenosis. J Emerg Med.

[5] Sobnosky S, Kohli R, Bleibel S (2006). Wellen's Syndrome. Int J Cardiol.

[6] Hovland A, Bjomstad H, Staub U, Vik-Mo H (2006). Reversible ischemia in Wellen's syndrome. J Nucl Cardiol.

[7] Tandy TK, Bottomy DP, Lewis JG (1999). Wellen's syndrome. Ann Emerg Med.

[8] Wellens HJJ, Conover MB (1992). The ECG in emergency decision making. WB Saunders Company.

[9] Elmenyar A (2000-2001). Wellens Syndrome. Heart Views.

[10] Riera A, Ferreira C, Filho C, Dubner S, Schapachnik E, Uchida A (2008). Wellens syndrome associated with prominent anterior QRS forces: An expression of left septal fascicular block?. J Electrocardiol.

